# Supplementary material for: Topical Application of Linezolid–Loaded Chitosan Nanoparticles for the Treatment of Eye Infections
Source: Nanomaterials (Basel). 2023 Feb 9;13(4):681. doi: 10.3390/nano13040681 (PMC9964951; doi:10.3390/nano13040681)
Supplement: Supplementary file 1 [file nanomaterials-13-00681-s001.zip › nanomaterials-2173684-supplementary.pdf]

Topical Application of Linezolid-loaded Chitosan Nanoparticles for the Treatment of Eye Infections

Musaed Alkholief [1], Mohd Abul Kalam [1], Abdullah Alshememry [1], Raisuddin Ali [1], Sulaiman S. Alhudaithi [1], Nasser B. Alsaleh [2], Mohammad Raish [1], Aws Alshamsan [1,*]

[1]*Department of Pharmaceutics, College of Pharmacy, King Saud University, PO Box-2457, Riyadh-11451, Saudi Arabia.*

[2]*Department of Pharmacology and Toxicology, College of Pharmacy, King Saud University, P.O. Box, 2457, Riyadh 11451, Saudi Arabia.*

malkholief@ksu.edu.sa (M.A); makalam@ksu.edu.sa (M.A.K); aalshememry@ksu.edu.sa (A.A); ramohammad@ksu.edu.sa (R.A) salhudaithi@ksu.edu.sa (S.S.H); nbalsaleh@ksu.edu.sa (N.B.A); mraish@ksu.edu.sa (M.R).

***** Correspondence: aalshamsan@ksu.edu.sa

Supplementary materials

Figure S1.

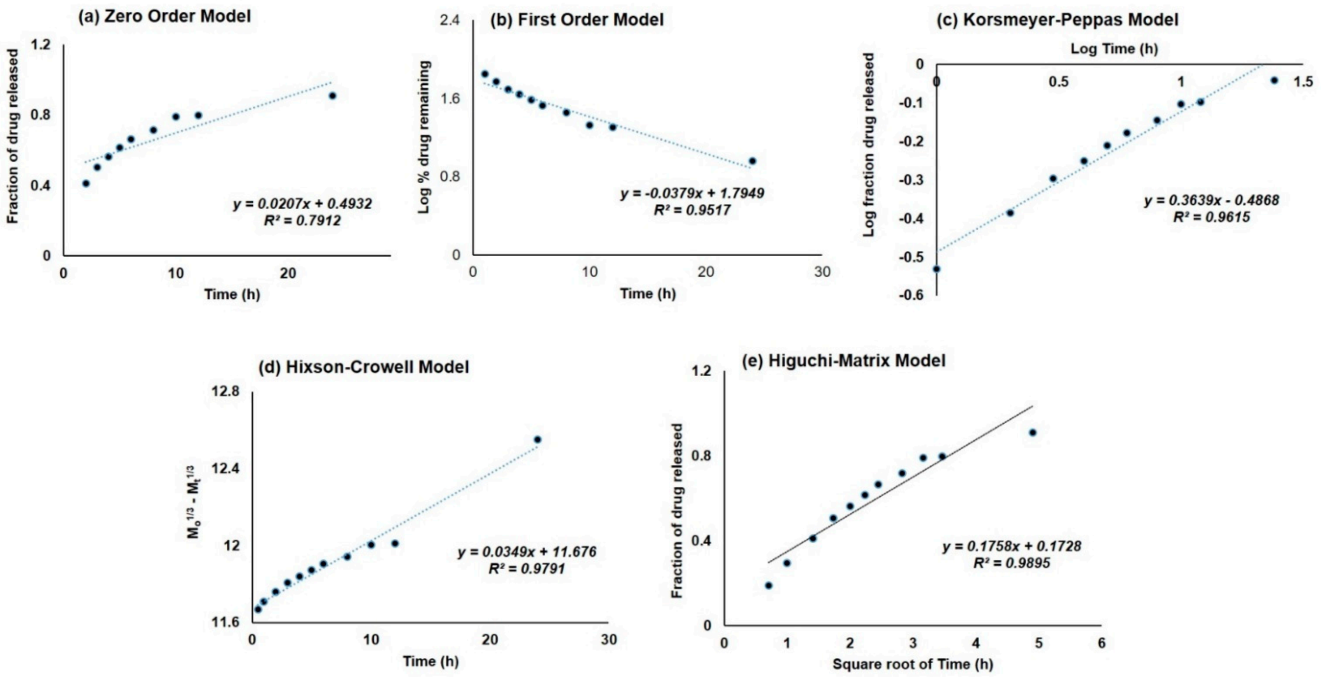

Figure S1. The plots of various kinetic models for the investigation of drug release kinetics of LZ-CSNPs. Where (a) Zero order; (b) First order; (c) Korsmeyer-Peppas; (d) Hixon-Crowell (e) Higuchi-Matrix.

Supplementary Tables

Table S1: Formulation development and preliminary characterizations. Data were presented as mean of three measurements with standard deviations.

Formulations	Size (nm) Mean ± SD	PDI (Mean ± SD)	ZP (mV) Mean ± SD	%EE (Mean ± SD)	%DL (Mean ± SD)
LZ-CSNPs-1 (20 mg TPP/81 mg CS)	113.6 ± 9.4	0.192 ± 0.046	28.2 ± 5.2	62.3 ± 5.6	10.4 ± 1.3
LZ-CSNPs-2 (78 mg TPP/162 mg CS)	209.7 ± 8.2	0.387 ± 0.134	23.1 ± 2.9	71.0 ± 5.2	11.2 ± 1.8
LZ-CSNPs-3 (32.5 mg TPP/94.5 mg CS)	223.6 ± 7.1	0.423 ± 0.121	23.6 ± 2.25	68.6 ± 8.5	11.1 ± 2.3

Table S2: Minimum inhibitory concentration (MIC) values of LZ-AqS and LZ-CSNPs against tested microorganisms. Data were presented as mean of three measurements with standard deviations.

Microorganisms	MICs of LZ-AqS (µg/mL) Mean ± SD
B. subtilis	5.33 ± 1.15
MRSA (SA 6538)	8.0 ± 2.0
S. aureus	5.33 ± 1.0
S. pneumoniae	4.0 ± 0.0
**Microorganisms**	**MIC of LZ-CSNPs (µg/mL) Mean ± SD**
B. subtilis	4.0 ± 2.0
MRSA (SA 6538)	6.67 ± 1.15
S. aureus	4.67 ± 1.15
S. pneumoniae	4.0 ± 0.0

Table S3 Grading system for ocular irritation test

**Cornea**		
**Lesion**		**Score**
**a. Opacity-Degree of density (area which is most dense is taken for reading)**		
No ulceration or opacity		**0**
Scattered or diffuse area – details of iris clearly visible		**1**
Easily discernible translucent areas, details of iris slightly obscured		**2**
Opalescent areas, no details of iris visible, size of pupil barely discernible		**3**
Opaque, iris invisible		**4**
**b. Area of cornea involved**		
One quarter (or less) but not zero		**1**
Greater than one quarter but less than one half		**2**
Greater than one half but less than three quarters		**3**
Greater than three quarters up to whole area		**4**
**Score equals (a x b x 5):**	**Total maximum =**	**80**
**Iris**		
**Lesion**		**Score**
**a. Values**		
Normal		**0**
Folds above normal, congestion, swelling, circumcorneal injection (any one or all of these or combination of any thereof), iris still reacting to light (sluggish reaction is		**1**
No reaction to light, hemorrhage; gross destruction (any one/ all of these)		**2**
**Score equals (a x 5):**	**Total maximum =**	**10**
**Conjunctiva**		
**Lesion**		**Score**
**a. Redness (refers to palpebral conjunctiva only)**		
Vessels normal		**0**
Vessels definitely injected above normal		**1**
More diffuse, deeper crimson red, individual vessels not easily discernible		**2**
Diffuse beefy red		**3**
**b. Chemosis**		
No swelling		**0**
Any swelling above normal (includes nictitating membrane)		**1**
Obvious swelling with partial eversion of the lids		**2**
Swelling with lids about half closed		**3**
Swelling with lids about half closed to completely closed		**4**
**c. Discharge**		
No any discharge		**0**
Any amount different from normal (does not include small amount observed in inner canthus of normal animals)		**1**
Discharge with moistening of the lids and hairs just adjacent to the lids		**2**
Discharge with moistening of the lids and considerable area around the eye		**3**
**Score equals (a + b + c) x 2:**	**Total maximum =**	**20**

Table S4: Classification of eye irritation scoring system

Classification of Irritation	Maximum Mean Total Score (MMTS*)
None	0.0-0.5
Practically none	0.6-2.5
Minimally	2.6-15.0
Mildly	15.1-25.0
Moderately	25.1-50.0
Severely	50.1-80.0
Extremely	80.1-100.0
Maximally	100.1-110.0
